# Anti-*Trypanosoma cruzi* activity of *Coptis* *rhizome* extract and its constituents

**DOI:** 10.1186/s41182-023-00502-2

**Published:** 2023-03-01

**Authors:** Yuki Tayama, Shusaku Mizukami, Kazufumi Toume, Katsuko Komatsu, Tetsuo Yanagi, Takeshi Nara, Paul Tieu, Nguyen Tien Huy, Shinjiro Hamano, Kenji Hirayama

**Affiliations:** 1 Department of Immunogenetics, Institute of Tropical Medicine (NEKKEN), Nagasaki University, 1-12-4 Sakamoto, Nagasaki, 852-8523 Japan; 2 Graduate School of Biomedical Sciences, Nagasaki University, 1-12-4 Sakamoto, Nagasaki, 852-8523 Japan; 3 Department of Immune Regulation, Institute of Tropical Medicine (NEKKEN), Nagasaki University, Nagasaki, Japan; 4 School of Tropical Medicines and Global Health, Nagasaki University, 1-12-4 Sakamoto, Nagasaki, 852-8523 Japan; 5 Section of Pharmacognosy, Institute of Natural Medicine, University of Toyama, Toyama, Japan; 6 NEKKEN Bio-Resource Center (NBRC), Institute of Tropical Medicine (NEKKEN), Nagasaki University, Nagasaki, Japan; 7 Faculty of Pharmacy, Iryo Sosei University, Iwaki, Fukushima Japan; 8 Faculty of Health Sciences, McMaster University, Hamilton, ON Canada; 9 Online Research Club, Nagasaki, Japan; 10 Department of Parasitology, Institute of Tropical Medicine (NEKKEN), Nagasaki University, Nagasaki, Japan; 11 The Joint Usage/Research Center On Tropical Disease, Institute of Tropical Medicine (NEKKEN), Nagasaki University, Nagasaki, 852-8523 Japan

**Keywords:** *Trypanosoma cruzi*, Chagas disease, *Coptis rhizome extract*, Coptisine chloride, Isoquinoline alkaloid

## Abstract

**Background:**

Current therapeutic agents, including nifurtimox and benznidazole, are not sufficiently effective in the chronic phase of *Trypanosoma cruzi* infection and are accompanied by various side effects. In this study, 120 kinds of extracts from medicinal herbs used for Kampo formulations and 94 kinds of compounds isolated from medicinal herbs for Kampo formulations were screened for anti-*T. cruzi* activity in vitro and in vivo.

**Methods:**

As an experimental method, a recombinant protozoan cloned strain expressing luciferase, namely Luc2-Tulahuen, was used in the experiments. The in vitro anti-*T. cruzi* activity on epimastigote, trypomastigote, and amastigote forms was assessed by measuring luminescence intensity after treatment with the Kampo extracts or compounds. In addition, the cytotoxicity of compounds was tested using mouse and human feeder cell lines. The in vivo anti-*T. cruzi* activity was measured by a murine acute infection model using intraperitoneal injection of trypomastigotes followed by live bioluminescence imaging.

**Results:**

As a result, three protoberberine-type alkaloids, namely coptisine chloride, dehydrocorydaline nitrate, and palmatine chloride, showed strong anti-*T. cruzi* activities with low cytotoxicity. The IC_50_ values of these compounds differed depending on the side chain, and the most effective compound, coptisine chloride, showed a significant effect in the acute infection model.

**Conclusions:**

For these reasons, coptisine chloride is a hit compound that can be a potential candidate for anti-Chagas disease drugs. In addition, it was expected that there would be room for further improvement by modifying the side chains of the basic skeleton.

**Supplementary Information:**

The online version contains supplementary material available at 10.1186/s41182-023-00502-2.

## Background

Chagas disease, caused by a protozoan parasite *Trypanosoma cruzi (T. cruzi)*, is a debilitating illness that affects from 6 to 7 million [1, 2] people mostly in Latin America. The disease is now expanding to non-endemic areas due to human migration [3]. The disease can be categorized into two distinct phases namely: an acute phase and a chronic phase. Acute phase is defined by high parasitemia, fever and lymphadenopathy and is usually resolved within 4–8 weeks [4]. Chronic phase begins after the acute phase and stays asymptomatic for decades (indeterminate phase) until clinical manifestation of the disease developed [3]. About 30–40% of chronically infected individuals develop typical clinical complications which involve cardiac and or gastrointestinal lesions [5].

Chemotherapeutics that are currently used for treatment of the disease are nifurtimox and benznidazole that have been used since 1960 [6, 7]. These drugs have been reported to show limited therapeutic activity against the infection [1] and have low compliance among patients due to the toxic side effects [8]. Still there is no vaccine for Chagas disease currently [9]. Thus, the lack of an efficient drug treatment requires the development of new anti-*T. cruzi* compound that has improved tolerability, safety, lower toxicity and improved efficacy on both phases of the disease [10].

Traditional Chinese medicine has more than 2000-year history and its standardization has been developed over a long period of time [11]. However, Kampo is a traditional treatment system originated from Chinese medicine was developed in Japan [12, 13]. In general, Kampo herbs are supposed to have a rich resource of active components [14, 15].

In this study, 120 kinds of extracts from medicinal herbs used for Kampo formulations and 94 kinds of compounds isolated from medicinal herbs for Kampo formulations which have been well quality controlled and maintained by Japanese leading research institute, Institute of Natural Medicine, University of Toyama, were screened for their anti-*T. cruzi* activity in vitro. We have confirmed that the protoberberine-type alkaloids with isoquinoline skeleton, which are the major active components of several plant extracts, showed a significant anti-*T. cruzi* activity in vitro as well as in vivo.

## Methods

### Kampo extracts and compounds

**Fig. 1:**
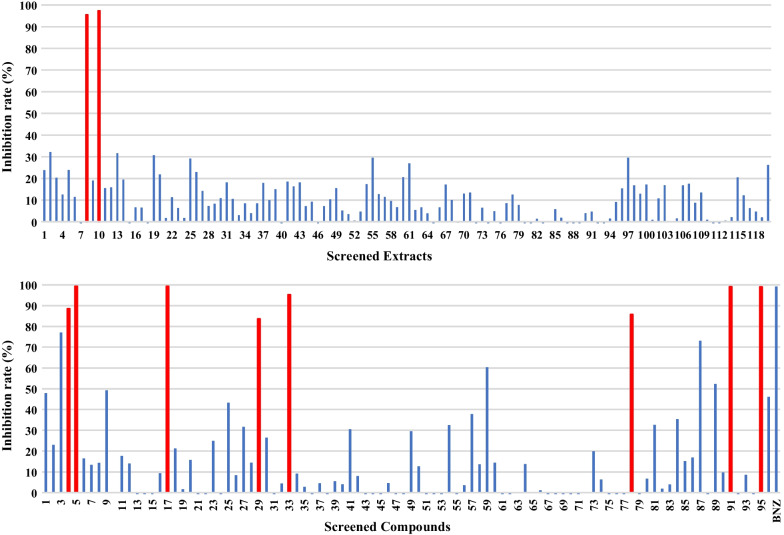
Screen of 120 kinds of extracts (20 μg/ml) and 94 kinds of compounds (20 μM) isolated from medicinal herbs by luminescence intensity to evaluate inhibitory effect. Data are presented as percent reduction in intensity. Experiments were done in triplicates

**Fig. 2:**
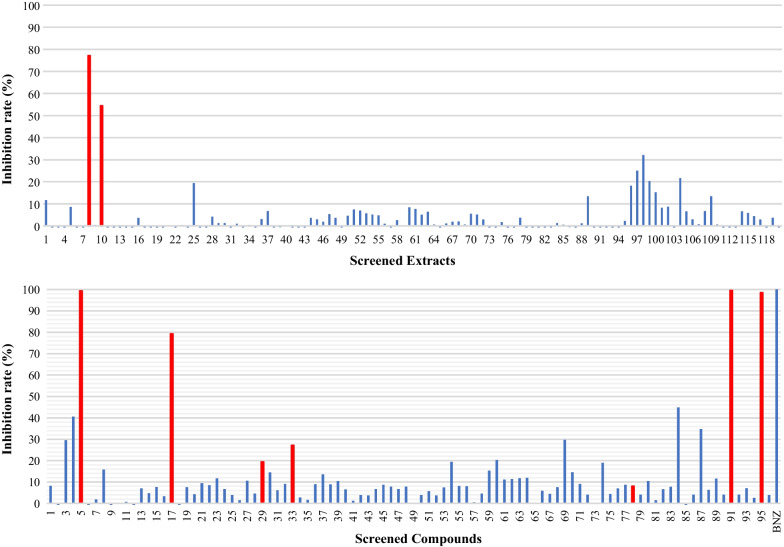
Cytotoxicity screen of 120 kinds of extracts (20 μg/ml) and 94 kinds of compounds (20 μM) isolated from medicinal herbs with alamarBlue reagent. Data are presented as percent reduction in intensity, indicating cell viability. Experiments were done in triplicates

The Kampo extracts and compounds library was provided by the Institute of Natural Medicine (The University of Toyama, Toyama, Japan). The library contains 120 kinds of extracts from medicinal herbs used for Kampo formulations and 94 kinds of compounds isolated from medicinal herbs for Kampo formulations (Additional file 1: Table S1, S2, Figs. 1, 2). Ultra-pure water generated by Milli-Q (Merck KGaA, Darmstadt, Germany) was the solvent for all herbal extracts and the concentration was adjusted by dry weight of the extract. Compounds were preserved at a concentration of 10 mM dissolved in dimethyl sulfoxide (DMSO; Wako Pure Chemicals Industrial Ltd, Japan). For more extensive experiments, coptisine chloride was purchased from Toronto Research Chemicals (Canada), and benznidazole was purchased from Sigma-Aldrich (USA). For in vivo administration solvent, 10 mg/ml solution in 7% Tween-80 (Sigma-Aldrich, USA), 3% ethanol (v/v) (Wako Pure Chemicals Industrial Ltd, Japan) and 90% (v/v) Milli-Q water [16] was prepared.

### Mammalian control and host cell lines

Newborn mouse heart fibroblast cells (NMH cells) were obtained from Bio-Resource Center, Institute of Tropical Medicine (NEKKEN), Nagasaki University. HuH28 (derived from 37-year-old female) cells [17] were maintained at Chulabhorn International College of Medicine, Thammasat University, Thailand.

### Parasites

We used a laboratory strain of *T. cruzi* which luciferase gene was transfected and integrated named Luc2-Tulahuen originally donated by Professor Takeshi Nara at Iryo Sosei University [18], and were obtained from the bio-resource center at the institute of tropical medicine (NEKKEN) funded by National Bio-resource Project (NBRP) Japan. Epimastigote form of *T. cruzi* was cultured in liver infusion tryptose (LIT) medium (Liver Infusion Broth (BD 226920), Tryptose (BD 211713), Becton Dickinson, USA) supplemented with 10% fetal bovine serum (FBS) (Gibco, USA), washed human red blood cells (Japan Red Cross Blood Center) and 500 mg/ml of G418 (Thermo Fisher Scientific, USA) at 26 °C until parasites reach to logarithmic stage. *T. cruzi* epimastigotes partially transformed to metacyclic forms were transferred to confluent NMH cells maintained in minimal essential medium (MEM) (Wako Pure Chemicals Industrial Ltd, Japan) supplemented with 10% newborn calf serum (Thermo Fisher Scientific, USA) at 37 °C and 5% CO_2_ as described elsewhere [19]. After 48 h incubation, free parasites outside the host cells were washed out and fresh complete MEM was added.

### In vitro anti-*T. cruzi* (trypomastigotes and amastigotes) assay

Luc2-Tulahuen trypomastigotes (3 × 10^4^/well) and NMH cells (5 × 10^4^/well) were mixed into a 96-well white plate. Test samples and controls were added and incubated with the cells for 72 h. NP-40 (Wako Pure Chemicals Industrial Ltd, Japan) was used as the positive control, and a solvent in which the drug was dissolved as a negative. After 72 h of incubation, 100 μl of luciferin solution containing 0.6% NP-40 (Piccagene) (Toyo Ink Group, Japan) was added. Luminescence intensity was measured by a plate reader (ARVO MX 1420) (Measurement time: 1 s) [20].

### In vitro anti-*T. cruzi* (intracellular amastigotes) assay

The intracellular amastigotes assay was described by Alonso-Padilla et al*.* [21]. Briefly, 6 × 10^6^ trypomastigotes and 3 × 10^6^ host cells (NMH cells) are seeded in a 25 cm^2^ flask in serum-free media (MEM + 1% newborn calf serum) to enhance the intracellular infection. After 24 h incubation, the cells were washed twice with phosphate buffered saline (PBS), then replaced by MEM + 10% newborn calf serum and incubated for another 24 h. Infected cells were detached by trypsinization. Infected host fibroblast cells (5 × 10^4^/well) were seeded into a 96-well plate. Test samples and the controls were added to the mixture and incubated for 72 h. Luminescence intensity was measured by a plate reader and the Piccagene (Measurement time: 1 s) [22, 23].

### In vitro assay for anti-epimastigote form of *T. cruzi* activity

Luc2-Tulahuen epimastigotes (2 × 10^5^/well) were dispensed into 96-well plate. After 72 h incubation, luminescence intensity was measured by adding lysis buffer with luciferin as substrate (PicaGene Luminescence Kit, Fuji Film Wako chemicals, Japan) (Measurement time: 1 s) [24].

The IC_50_ was calculated using the following equation:

$${\mathrm{IC}}_{50}{=10}^{\mathrm{log}\left(\frac{\mathrm{A}}{\mathrm{B}}\right)\times \frac{50-C}{\mathrm{D}-C}+\mathrm{log}\left(\mathrm{B}\right)},$$

where A is the lowest concentration at which the percentage inhibition exceeds 50%, B is the highest concentration at which percentage inhibition is less than 50%, and C and D are the percentage inhibition of the sample at concentrations B and A, respectively.

### Evaluation of cytotoxicity and measurement of 50% injury concentration

NMH cells or HuH28 cells (1 × 10^4^/well) of 100 μl aliquots were seeded on a 96-well black plate. Test samples were added and incubated for 72 h. NP-40 was added as a positive control (100% cytotoxicity) and a solvent in which the samples were dissolved as a negative control (0% cytotoxicity). Then 10 μl of alamarBlue reagent (10%, Funakoshi Co., Tokyo, Japan) for mitochondria staining was added and incubated for another 4 h. The fluorescence intensity (544 nm/590 nm) was measured with a plate reader (measurement time: 0.1 s).

The concentration required to reduce cell viability by 50% (CC_50_) was calculated using the following equation:

$${\mathrm{CC}}_{50}{=10}^{\mathrm{log}\left(\frac{\mathrm{A}}{\mathrm{B}}\right)\times \frac{50-C}{\mathrm{D}-C}+\mathrm{log}\left(\mathrm{B}\right)},$$

where A is the lowest concentration at which the cell viability exceeds 50%, B is the highest concentration at which cell viability is less than 50%, and C and D are the cell viability of the sample at concentrations B and A, respectively.

### Infection of mice

Mice were housed and maintained in Nagasaki University Biomedical Research Center (12 h light/dark cycle). Female BALB/c mice from 6 to 10 weeks old (20–25 g) were used in all the experiments. Mice were obtained as wild type from SLC (Japan). In standard experiments, 5 × 10^3^ in vitro tissue culture-derived trypomastigotes (TCTs) was intra-peritoneally (i.p.) inoculated into mouse [25–27]. The mice were handled according to the international guidelines and institutional guideline of Nagasaki University for the use and maintenance of experimental animals. Ethical approval for this study was obtained from the institutional ethical review board, Nagasaki University (approval number R12005).

### Bioluminescence imaging for efficacy evaluation

Mice were intra-peritoneally injected with 150 mg/kg d-luciferin (SYD labs, USA), then anesthetized using 2.5% (vol/vol) gaseous isoflurane in oxygen. To measure bioluminescence, mice were placed in an IVIS Lumina II system (Caliper Life Science, USA) and images were acquired 10 min after d-luciferin administration using LivingImage 4.3 (Caliper Life Sciences, USA). Exposure time was fixed as 5 min. Anesthesia was maintained throughout the imaging process through the nose cone. Whole body luminescence was determined by drawing the region of interest (ROI) and quantifying the bioluminescence expressed as total flux (photons/second; p/s) [16, 28]. Coptisine chloride was administered intra-peritoneally (i.p.) at a dose of 30 mg/kg twice a day. Benznidazole (100 mg/kg) as a positive control and the drug solvent as a negative control were administered orally (p.o.) once a day. Drug was administered for 5 consecutive days from day 4 to 8 of infection. Measurements with IVIS Lumina II were performed at five different times: on day 3 of infection (the day before drug treatment), on day 9 of infection (the day after drug treatment was completed), on day 14, 28 and 40 of infection. To maintain fairness, different experimenters performed drug treatments and luminescence intensity measurements. At day 29, BALB/c mice were injected with cyclophosphamide (Sigma-Aldrich, USA) (200 mg/kg) as an immunosuppressant by i.p. and were followed by a maximum of 3 doses at 3 day intervals [16, 29]. The use of immunosuppressant like cyclophosphamide could help enhance the visibility of the infected lesions even after 100 dpi. In this animal study, treatment was given when the luminescence intensity was high. In addition, an immunosuppressant was administered when the difference in luminescence intensity among the three groups became small due to natural immunity of the mice.

### Statistical analysis

Data were tabulated on Microsoft Excel and statistically analyzed. For statistical comparisons, ANOVA analysis was used to determine the statistical significance of difference in values from the control groups. Data were expressed as the mean ± standard error, and the results were obtained from at least three independent experiments. A *p*-value < 0.05 was considered statistically significant.

## Results

### In vitro assay

One hundred and twenty extracts and 94 compounds were screened for their cytotoxic effect on the trypomastigotes and amastigotes using a mixture culture experiment (Fig. 1), some sample were shown to exhibit more than 80% reduction of parasite signals. Those positive samples were Phellodendron bark (the bark of *Phellodendron amurense Ruprecht*) and Coptis rhizome (the rhizome of *Coptis japonica Makino, Coptis chinensis Franchet, Coptis deltoidea C.Y. Cheng et Hsiao or Coptis teeta Wallich*) from the extracts, Alison B, Alkanin, Berberin chloride, Coptisine chloride, Dehydrocorydaline nitrate, Palmatine chloride, Shikonin and Timosaponin A-III from the compounds indicated by red colored in Fig. 1.

Similar set of the Kampo library was applied to mouse fibroblast cells (NMH) and human bile duct carcinoma cell line (HuH28) to observe any cytotoxic effect on host cells as shown in Fig. 2. Within two extract samples (Phellodendron bark, Coptis rhizome) and 8 compounds samples (Alison B, Alkanin, Berberin chloride, Coptisine chloride, Dehydrocorydaline nitrate, Palmatine chloride, Shikonin, Timosaponin A-III) which showed more than 80% inhibition in the *T. cruzi* mixture culture (Fig. 1), only three samples (Coptisine chloride, Dehydrocorydaline nitrate, Palmatine chloride) showed relatively lower cytotoxicity (Fig. 2). Therefore, we decided to select those three positive compounds (Coptisine chloride, Dehydrocorydaline nitrate, Palmatine chloride) for further analysis. Those three compounds shared the same isoquinoline skeleton namely coptisine chloride, dehydrocorydaline nitrate, and palmatine chloride.

**Fig. 3:**
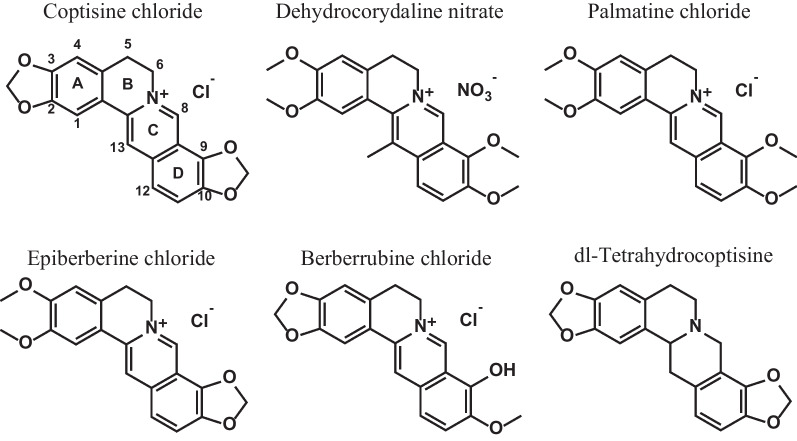
Chemical structure of the investigated compounds

After the first screening as shown in Figs. 1 and 2 as a representative of three repeated experiments, we determined IC_50_ and CC_50_ values of the selected candidate compounds. As performed in the first in vitro screening, we used two *T. cruzi-*culture systems for IC_50_ as shown in Table 1. For this second screening, we added three more compounds, epiberberine chloride, berberrubine chloride, dl-tetrahydro coptisine to already selected compounds belonging to the same protoberberine-type alkaloids (Fig. 3, Table 1).

**Table 1:** IC_50_, and CC_50_, of different compounds on *T. cruzi* (Trypo: trypomastigote, Ama: amastigote, Epi: epimastigote) and mammalian cell (NMH, HuH28) lines

Cells	*T. cruzi* Trypo + Ama	*T. cruzi* Intracell Ama	*T. cruzi* Epi	NMH	HuH28
Assay	Luciferase activity			AlamarBlue viability	
	IC_50_ (μM)			CC_50_ (μM)	
Coptisine chloride	4.48	4.98	15.1	> 40	> 100
Dehydrocorydaline nitrate	3.47	7.89	17.3	> 40	> 100
Palmatine chloride	2.66	17.4	22.3	> 40	> 100
Epiberberine chloride	> 40	> 40	NT	> 40	> 100
Berberrubine chloride	20.1	33.5	NT	> 40	> 100
dl-Tetrahydro coptisine	> 40	> 40	NT	> 40	> 100
Benznidazole	2.78	4.91	3.78	> 40	> 100

Each experiment was repeated three times and representative data were shown

The reduced luminescence intensity of luciferin-expressing protozoa suggested that coptisine chloride (IC_50_: 4.48 μM), dehydrocorydaline nitrate (IC_50_: 3.47 μM) and palmatine chloride (IC_50_: 2.66 μM) had an antiprotozoan effect (trypomastigotes in the medium or intracellular amastigotes). It was found to be as effective as benznidazole (IC_50_: 2.78 μM) in activity quantification (Additional file 1: Fig. S1, Table S1).

Next, the effect on proliferative amastigotes in trypanosomes infected with NMH cells was estimated. Coptisine chloride (IC_50_: 4.98 μM) was the most effective as benznidazole (IC_50_: 4.91 μM) due to reduced luminescence intensity in luciferin-expressing protozoa (Additional file 1: Fig. S2, Table S1). On the other hand, coptisine chloride (IC50: 15.1 µM) was fourfold less effective as benznidazole (IC50: 3.78 µM) against epimastigotes (Additional file 1: Fig. S3, Table S1). Cytotoxicity to NMH cells or HuH28 cells was examined for those 6 compounds (Additional file 1: Figs S4, S5). These results indicate that there are differences in the effectiveness of the isoquinoline skeleton depending on the number of methoxy groups and side chains (Fig. 3, Table 1).

### In vivo assay

**Fig. 4:**
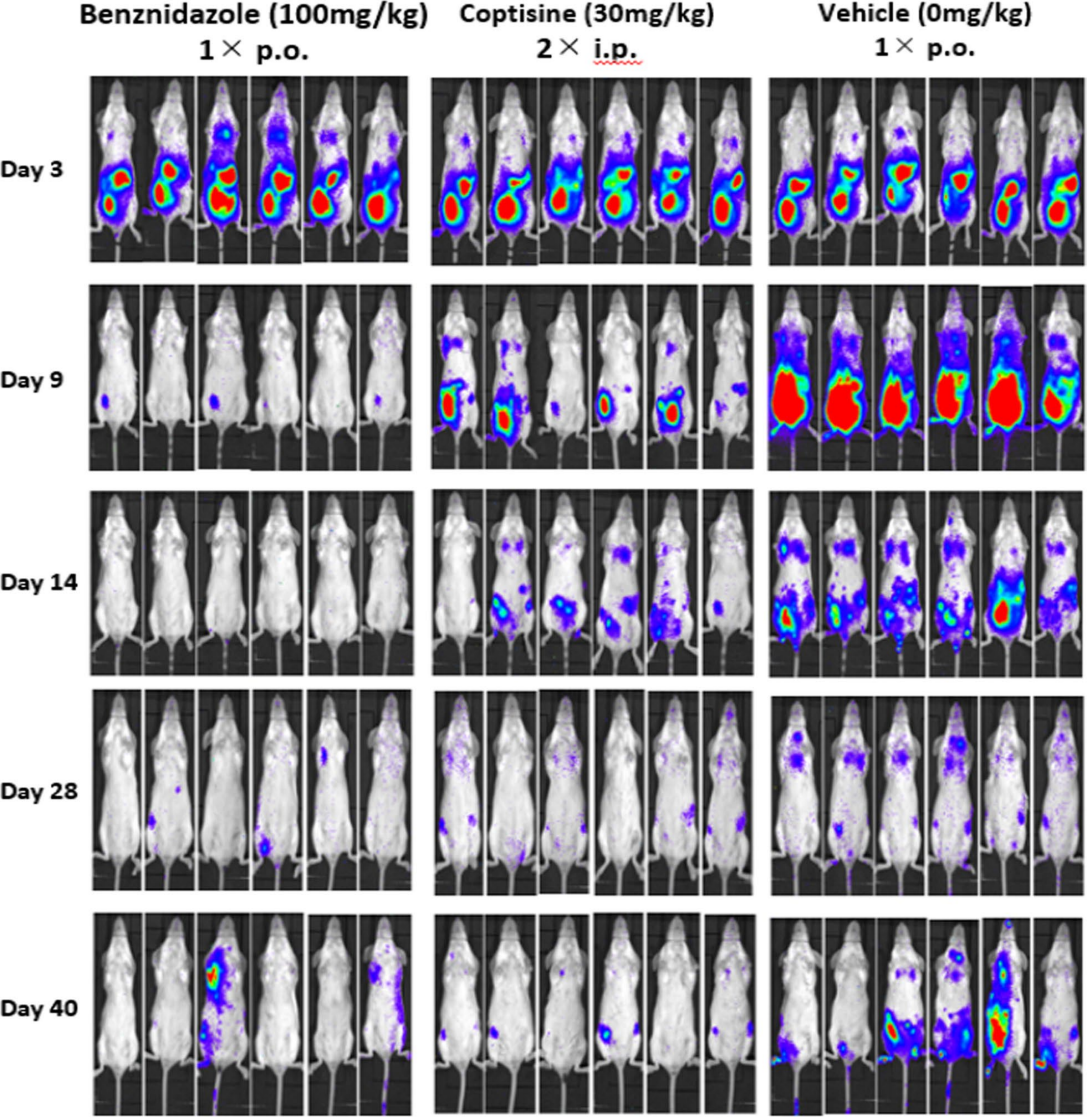
Female BALB/c mice infected with 5 × 10^3^ trypomastigotes were treated with coptisine chloride (30 mg/kg, 2 × i.p.); positive control is benznidazole (100 mg/kg, 1 × p.o.); negative control is the drug solvent. Bioluminescence imaging was obtained in an IVIS Lumina II system

**Fig. 5:**
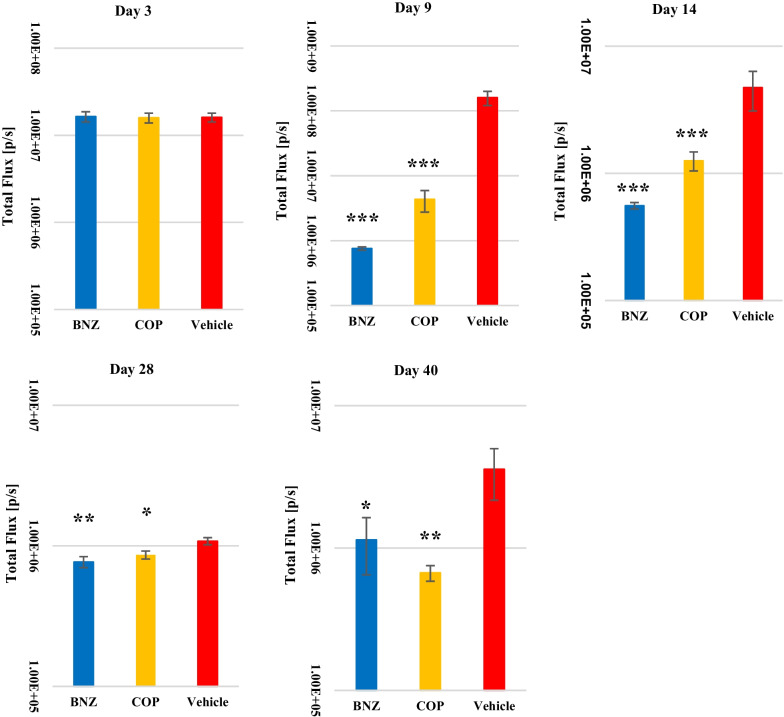
Luminescence intensity at different timepoints in the mice model infected with 5 × 10^3^ trypomastigotes and treated with coptisine chloride (COP) (30 mg/kg, 2 × i.p.); positive control is benznidazole (BNZ) (100 mg/kg, 1 × p.o.); negative control is the drug solvent. Bioluminescence imaging was obtained in an IVIS Lumina II system. Data are presented as mean and standard deviation in intensity of three experiments (*p < 0.05, **p < 0.01, ***p < 0.005 when compared with vehicle). In this experiment, we excluded mice with luminescence intensity less than 1 × 10^7^ in pre-drug measurements (Day 3)

After a 5-day treatment regimen with coptisine chloride (30 mg/kg bid i.p.) from day 4 to 8, the luminescence intensity was significantly reduced in the treated group compared with the untreated group. Though the luminescence intensity of the coptisine chloride group was slightly higher than that of the benznidazole group, still significant reduction was observed, as shown in Figs. 4 and 5. It was found that the luminescence intensity of the coptisine chloride-treated group was significantly reduced even after the immune suppression treatment (day 40) compared with the control group (Figs. 4, 5). Therefore, it was shown that coptisine chloride has a therapeutic effect on the acute phase in vivo model.

## Discussion

Chagas disease is expanding to non-endemic areas and an effective treatment is needed. Herbal compounds from traditional medicine are promising candidates for drug development, and their efficacy was demonstrated in our study.

The experimental system of mixing trypomastigotes and NMH cells confirmed the effects on trypomastigotes in the medium and amastigotes in the cells. The reduced luminescence intensity of luciferin-expressing protozoa suggested that coptisine (IC_50_: 4.48 μM), dehydrocorydaline (IC_50_: 3.47 μM) and palmatine (IC_50_: 2.66 μM) had an antiprotozoal effect. It was found to be as effective as benznidazole (IC_50_: 2.78 μM) in activity quantification (Table 1). The experiment mimics the acute phase of the disease with trypomastigotes in the blood and amastigotes in the cells [30, 32]. As a result, these compounds are thought to be effective in the acute phase. In addition, Phellodendron bark and Coptis rhizome showed more than 80% inhibition in the T. cruzi mixture culture (Fig. 1). However, these extracts also showed cytotoxicity. These consist of berberine, coptisine and palmatine. The cytotoxicity of Phellodendron bark and Coptis rhizome may be attributed to berberine.

However, the urgent requirement from the present clinical setting in the world is to get an effective curative drug for chronic phase infection [1–3]. Therefore, future work will focus on testing the Kampo compounds in the chronic stage of infection.

The experimental system for intracellular amastigotes using NMH cells allowed us to confirm the effect on proliferative amastigotes in trypanosomes-infected NMH cells which mimics the chronic phase of the disease [31, 32]. Coptisine (IC_50_: 4.98 μM) showed comparable levels of IC_50_ as benznidazole (IC_50_: 4.91 μM) (Table 1). Although we have not yet examined in vivo chronic Chagas model, coptisine was revealed to be a possible candidate for an effective drug in the chronic phase.

As shown in Table 1, other related protoberberine-type alkaloids, dehydrocorydaline and palmatine showed strong activity in the mixture experiment with trypomastigote, amastigote and NMH, but not against intracellular amastigotes. When those three active compounds were examined for their cytotoxicity on epimastigote, they showed almost equal effectiveness of IC_50_ around 15–20 μM, which were less effective compared with benznidazole (3.78 μM). Therefore, coptisine is specifically more active to amastigote than to epimastigote, whereas benznidazole shows both stages active. In addition, three active protoberberine-type alkaloids showed equal cytotoxicity on the *T. cruzi* epimastigote cells, but the effect on the intracellular amastigote significantly increased in only coptisine which might be due to the increased permeability through host cell membrane.

Although comparison on the activities of tested compounds did not show clear structure–activity relationships, following trends were observed. The presence of methylenedioxy at rings A (at C-2 and 3) and D (C-9 and 10) and aromatization of ring C could be the key factor for the anti-*Trypanosoma cruzi* activity. However, the effects of the presence of methylenedioxy or methoxy groups of rings A/D were not clear. The presence of methyl group at C-13 may reduce the anti-*Trypanosoma cruzi* activity on the intracellular amastigote. The presence of a hydroxy group at C-9 may reduce the activity. To clarify the structure–activity relationships of the protoberberine-type alkaloids on anti-*Trypanosoma cruzi* activity, further studies using more diverse derivatives are required.

In animal experiments, we confirmed the equivalent parasiticidal activity of coptisine as benznidazole as evidenced by the reduction in luminescence intensity shown in Figs. 4 and 5. In addition, the relapse detected on day 40 after administration of the immunosuppressant during a period between day 30 and 39 was smaller than that in the benznidazole treatment group. This suggests that coptisine was more effective to reduce the number of parasites compared with the current standard curative protocol of benznidazole in the acute model [28]. Taken together, coptisine is a novel anti-*Trypanosoma cruzi* compound with an equivalent effectiveness as benznidazole when examined in vitro acute and chronic and by in vivo acute model. Our results also indicate that berberine structure with an isoquinoline skeleton might be used for more effective compounds design by the modification of side chains.

It has been reported that coptisine chloride or protoberberine-type alkaloids has various pharmaceutical effects such as anti-cancer [36, 37], anti-inflammation, and anti-diabetic as well as anti-infectious diseases [33–35, 38, 39]. Coptisine non-competitively inhibits DHODH in *Plasmodium falciparum* and showed weak inhibitory activity against human DHODH [35]. *T. cruzi* DHODH, which catalyzes the production of orotate, and was demonstrated to be essential for *T. cruzi* survival, could be one of our coptisine’s potential targets [40–42].

It has been reported that coptisine chloride possesses anti-cancer activity through the inhibition of PI3K/Akt/mTOR signaling and subsequent mitochondrial ROS production in the hepatocellular cancer Hep3B cells [36, 37]. *T. cruzi* infection has been demonstrated to stimulate host PI3K signaling in human and mice macrophages that allow intracellular parasites to grow and survive [43–47]. These reports suggest that inhibition of PI3K/Akt/mTOR signaling by coptisine chloride may have affected the growth and survival of intracellular parasites. Coptisine chloride has also been reported to inhibit LPS-stimulated inflammation by blocking the activation of NF-κB and MAPK in macrophages [48–51]. In coptisine chloride therapy for colitis, coptisine chloride significant suppresses mRNA expression, releases of pro-inflammatory cytokines (TNF-α, IFN-γ, IL-1β, IL-6, IL-17) and enhances the mRNA expression level of IL-10, an anti-inflammatory cytokine [52, 53].

*T. cruzi* infection could stimulate both protective and pathogenic host immune responses through natural or adaptive immunological pathways [54–59]. Although we did not see any significant adverse events during in vivo experiment, previously reported immune inhibitory effects of coptisine should be carefully observed.

When considering drug development, the target product profile (TPP) is important, and oral administration is preferable for aiming a therapeutic drug for chronic Chagas disease that may need at least 2 weeks regimen. However, it is known that the oral bioavailability of coptisine chloride is low [60]. It is necessary to investigate whether these problems can be solved by improving the side chain or enclosing the compound to improve the blood concentration.

In the present study, we identified a series of protoberberine-type alkaloids as strong candidate anti*-Trypanosoma cruzi* medicine using in vitro and in vivo models. Among them, coptisine was expected to be the most effective candidate compound.

The model mouse used this time has poor persistence of luminescence intensity, and it is considered difficult to measure in the chronic phase. However, it may be possible to measure the chronic phase using new recombinant protozoa. In this study, we found that compounds identified in vitro are also effective in vivo. Since chronic Chagas treatment is a typical neglected tropical disease unmet needs [61, 62], further development must be facilitated.

## Supplementary Information

**Additional file 1.** Supplementary tables and figures.

## Data Availability

The datasets used and/or analyzed during the current study are available from the corresponding author on reasonable request.

## Abbreviations

IC50: Half-maximal inhibitory concentration
DMSO: Dimethyl sulfoxide
NMH: Newborn mouse heart fibroblast
IP: Intraperitoneal injection
PO: Per OS
PBS: Phosphate buffered saline
NP40: Nonyl phenoxypolyethoxylethanol 40
LIT: Liver infusion tryptose
FBS: Fetal bovine serum
NBCS: Newborn calf serum
MEM: Minimum essential medium
TCTs: Tissue culture-derived trypomastigotes
BNZ: Benznidazole
COP: Coptisine chloride
