# Supplementary material for: Anti-Trypanosoma cruzi activity of Coptis rhizome extract and its constituents
Source: Trop Med Health. 2023 Mar 1;51:12. doi: 10.1186/s41182-023-00502-2 (PMC9976467; doi:10.1186/s41182-023-00502-2)
Supplement: Supplementary file 1 — Additional file 1. Supplementary tables and figures. [file 41182_2023_502_MOESM1_ESM.docx]

**Supplementary Information**

**Screening of a library of Kampo extracts and compounds from medicinal herbs to identify anti-Trypanosoma cruzi**

Yuki Tayama^1,2^, Shusaku Mizukami^3,4, 11^, Kazufumi Toume^5^, Katsuko Komatsu^5^, Tetsuo Yanagi^6, 11^, Takeshi Nara^7^, Paul Tieu^8,9^, Nguyen Tien Huy^1,4,9^, Shinjiro Hamano^2,10, 11^, Kenji Hirayama^1,2,4, 11*^

^1^Department of Immunogenetics, Institute of Tropical Medicine (NEKKEN),

Nagasaki University, 1-12-4 Sakamoto, Nagasaki, 852-8523, Japan

^2^Graduate School of Biomedical Sciences, Nagasaki University, 1-12-4 Sakamoto, Nagasaki, 852-8523, Japan

^3^Department of Immune Regulation, Institute of Tropical Medicine (NEKKEN), Nagasaki University, Nagasaki, Japan

^4^School of Tropical Medicines and Global Health, Nagasaki University, 1-12-4 Sakamoto, Nagasaki, Japan

^5^Section of Pharmacognosy, Institute of Natural Medicine, University of Toyama, Toyama, Japan

^6^NEKKEN Bio-Resource Center (NBRC), Institute of Tropical Medicine (NEKKEN), Nagasaki University, Nagasaki, Japan

^7^Faculty of Pharmacy, Iryo Sosei University, Iwaki, Fukushima, Japan

^8^Faculty of Health Sciences, McMaster University, Ontario, Canada

^9^Online Research Club (https://www.onlineresearchclub.org/), Nagasaki, Japan

^10^ Department of Parasitology, Institute of Tropical Medicine (NEKKEN), Nagasaki University, Nagasaki, Japan

^11^ The Joint Usage/Research Center on Tropical Disease, Institute of Tropical Medicine (NEKKEN), Nagasaki University, Nagasaki 852-8523, Japan

*Corresponding author: Kenji Hirayama, School of Tropical Medicines and Global Health, Nagasaki University, 1-12-4 Sakamoto, Nagasaki, 852-8523, Japan. Telephone: +81 (0) 958197949.

Email: [hiraken@nagasaki-u.ac.jp](mailto:hiraken@nagasaki-u.ac.jp)

**List of contents**

**Supplement Table 1.** List of extracts in Kampo library.

**Supplement Table 2.** List of compounds in Kampo library.

**Supplement Figure 1.** IC_50_ curve of the 6 compounds derived from herbal drugs targeting infecting NMH cells and trypomastigotes. No loss of intensity was observed for dl-tetrahydrocoptisine.

**Supplement Figure 2.** IC_50_ curve of the 6 compounds derived from herbal drugs targeting infecting NMH cells include in amastigotes. No loss of intensity was observed for epiberberine and dl-tetrahydrocoptisine.

**Supplement Figure 3.** IC_50_ curve of the 3 compounds derived from herbal drugs targeting epimastigotes.

**Supplement Figure 4.** Cytotoxicity assay of 6 compounds derived from herbal drugs against normal NMH cells. Berberrubine chloride had a slight cytotoxic effect.

**Supplement Figure 5.** Cytotoxicity assay of 6 compounds derived from herbal drugs against normal HuH28 cells. None showed cytotoxicity.

| ID.No. | Crude drug extract name | ID.No. | Crude drug extract name | ID.No. | Crude drug extract name |
| --- | --- | --- | --- | --- | --- |
| 1 | Clematis root | 41 | Asiasarum root | 81 | Citrus unshiu peel |
| 2 | Artemisia capillaris flower | 42 | Saffron | 82 | Gastrodia tuber |
| 3 | Turmeric | 43 | Smilax rhizome | 83 | Asparagus root |
| 4 | Lindera root | 44 | Gardenia fruit | 84 | Benincasa seed |
| 5 | Corydalis tuber | 45 | Panax notoginseng Root | 85 | Angelica dahurica root |
| 6 | Astragalus root | 46 | Cornus fruit | 86 | Codonopsis root |
| 7 | Scutellaria root | 47 | Japanese zanthoxylum peel | 87 | Peach kernel |
| 8 | Phellodendron bark | 48 | Jujube seed | 88 | Aralia rhizome |
| 9 | Cherry bark | 49 | Dioscorea rhizome | 89 | Eucommia bark |
| 10 | Coptis rhizome | 50 | Sparganium rhizome | 90 | Cistanche herb |
| 11 | Polygala root | 51 | Rehmannia root | 91 | Ginseng |
| 12 | Artemisiae leaf | 52 | Eleutherococcus senticosus rhizome | 92 | Fritillaria bulb |
| 13 | Polygonum root | 53 | Lycium Bark | 93 | Densefruit Pittany Root Bark |
| 14 | Curcuma Rhizome | 54 | Tribulus fruit | 94 | Ophiopogon root |
| 15 | Pueraria root | 55 | Peony root | 95 | Mentha herb |
| 16 | Trichosanthes root | 56 | Plantago seed | 96 | Pinellia tuber |
| 17 | Processed ginger | 57 | Processed rehmannia root | 97 | Japanese angelica root |
| 18 | Glycyrrhiza | 58 | Amomum seed | 98 | Atractylodes rhizome |
| 19 | Platycodon root | 59 | Ginger | 99 | Loquat leaf |
| 20 | Chrysanthemum flower | 60 | Cimicifuga rhizome | 100 | Areca |
| 21 | Immature orange | 61 | Magnolia flower | 101 | Poria sclerotium |
| 22 | Notopterygium | 62 | Gentiana macrophylla root | 102 | Sinomenium stem and rhizome |
| 23 | Apricot kernel | 63 | Red peony root | 103 | Imperata rhizome |
| 24 | Sophora root | 64 | Cnidium rhizome | 104 | Saposhnikovia root and rhizome |
| 25 | Sasa leaf | 65 | Ginseng | 105 | Processed aconite root |
| 26 | Schizonepeta spike | 66 | Atractylodes lancea rhizome | 106 | Quercus bark |
| 27 | Suberect spatholobus tem | 67 | Mulberry bark | 107 | Malaytea scurfpea fruit |
| 28 | Cinnamom Bark | 68 | Mulberry Leaf | 108 | Moutan Bark |
| 29 | Scrophularia root | 69 | Dipsacus root | 109 | Ephedra herb |
| 30 | Safflower | 70 | Perilla herb | 110 | Hemp fruit |
| 31 | Silktree albizia bark | 71 | Rhubarb | 111 | Shrub chaste tree fruit |
| 32 | Red Ginseng | 72 | Jujube | 112 | Saussurea root |
| 33 | Cyperus rhizome | 73 | Alisma tuber | 113 | Myrrh |
| 34 | Magnolia bark | 74 | Salvia miltiorrhiza root | 114 | Bitter cardamon |
| 35 | Achyranthes root | 75 | Bamboo culm | 115 | Leonurus herb |
| 36 | Euodia fruit | 76 | Panax japonicus rhizome | 116 | Coix seed |
| 37 | Burdock fruit | 77 | Anemarrhena rhizome | 117 | Japanese gentian |
| 38 | Sesame | 78 | Clove | 118 | Alpinia officinarum rhizome |
| 39 | Schisandra fruit | 79 | Uncaria hook | 119 | Ganoderma |
| 40 | Bupleurum root | 80 | Polyporus sclerotium | 120 | Forsythia fruit |

**Supplement Table 1.** List of crud drug extracts in Kampo library.

| ID.No. | Compound name | ID.No. | Compound name | ID.No. | Compound name |
| --- | --- | --- | --- | --- | --- |
| 1 | Acontine | 33 | Dehydrocorydaline nitrate |  |  |
| 2 | Albiflorin | 34 | Dehydrocostuslactone | 66 | Limonin |
| 3 | Alisol A | 35 | Demethoxycurcumine | 67 | Liquiritin |
| 4 | Alison B | 36 | Dihydrocapsaicin | 68 | Loganin |
| 5 | Alkanin | 37 | *Dimethylsculetin* | 69 | Luteolin |
| 6 | Amygdalin | 38 | Eleutheroside B | 70 | Magnolol |
| 7 | Arbutin | 39 | (-)-Epigallocathechin gallate | 71 | Mesaconitine |
| 8 | Astragaloside IV | 40 | Epihesperidine | 72 | Naringin |
| 9 | Atractylenolide III | 41 | Ergosterol | 73 | Nodakenin |
|  |  | 42 | β- Eudesmol | 74 | Osthol |
| 11 | Atropin sulphate | 43 | Evodiamine | 75 | Oxymatrine |
| 12 | Aucubin | 44 | (*E*)-Ferulic acid | 76 | Paeoniflorin |
| 13 | Baicalein | 45 | Geniposide | 76 | Paeoniflorin |
| 14 | Baicalin | 46 | Geniposidic acid | 77 | Paeonol |
| 15 | Barbaloin | 47 | Gentiopicroside | 78 | Palmatine chloride |
| 16 | Benzoylmesaconine hydrochloride | 48 | (6)-Gingerol | 79 | Perillaldhyde |
| 17 | Berberin chloride | 49 | Ginsenoside-Rb1 | 81 | Puerarin |
| 18 | Bergenin | 50 | Ginsenoside-Rc | 82 | Rhynchophylline |
| 19 | Bisdemethoxycurcumin | 51 | Ginsenoside-Rd | 83 | Rosmaric Acid |
| 20 | Bufalin | 52 | Ginsenoside-Re | 84 | Saikosaponin a |
| 21 | Bufotalin | 53 | Ginsenoside-Rg1 | 85 | Saikosaponin b2 |
| 22 | Cappillarisin | 54 | Glabridine | 86 | Saikosaponin c |
| 23 | (*E*)-Capsaicin | 55 | Glycyrrhizic acid | 87 | Saikosaponin d |
| 24 | Catalpol | 56 | Gomison A | 88 | Schizandrin |
| 25 | (*E*)-Chlorogenic acid | 57 | Gomison N | 89 | Sennoside A |
| 26 | (*E*)-Cinnamic acid | 58 | Hesperidin | 90 | Sennoside B |
| 27 | Cinobufagin | 59 | Hirsutine | 91 | Shikonin |
| 28 | Cinobufotalin | 60 | Honokiol | 92 | (6)-Shogaol |
| 29 | Coptisine chloride | 61 | Hypacontine | 93 | Sinomenine |
| 30 | Corydalin | 62 | Icariin | 94 | Swertiamarin |
| 31 | Costunolide | 63 | Isofraxidine | 95 | Timosaponin A-III |
| 32 | Curcumin | 64 | Isorhynchophyllin | 96 | Wogonin |

**Supplement Table 2.** List of compounds in Kampo library.

**Supplement Figure 1.** IC_50_ curve of the 6 compounds derived from herbal drugs targeting infecting NMH cells and trypomastigotes. No loss of intensity was observed for dl-tetrahydrocoptisine.

**Supplement Figure 2.** IC_50_ curve of the 6 compounds derived from herbal drugs targeting infecting NMH cells include in amastigotes. No loss of intensity was observed for epiberberine and dl-tetrahydrocoptisine.

**Supplement Figure 3.** IC_50_ curve of the 3 compounds derived from herbal drugs targeting epimastigotes.

**Supplement Figure 4.** Cytotoxicity assay of 6 compounds derived from herbal drugs against normal NMH cells. Berberrubine chloride had a slight cytotoxic effect.

**Supplement Figure 5.** Cytotoxicity assay of 6 compounds derived from herbal drugs against normal HuH28 cells. None showed cytotoxicity.
